# Supplementary material for: Increased HIF-2α activity in the nucleus pulposus causes intervertebral disc degeneration in the aging mouse spine
Source: Front Cell Dev Biol. 2024 Mar 6;12:1360376. doi: 10.3389/fcell.2024.1360376 (PMC10950937; doi:10.3389/fcell.2024.1360376)
Supplement: Supplementary file 1 [file DataSheet1.pdf]

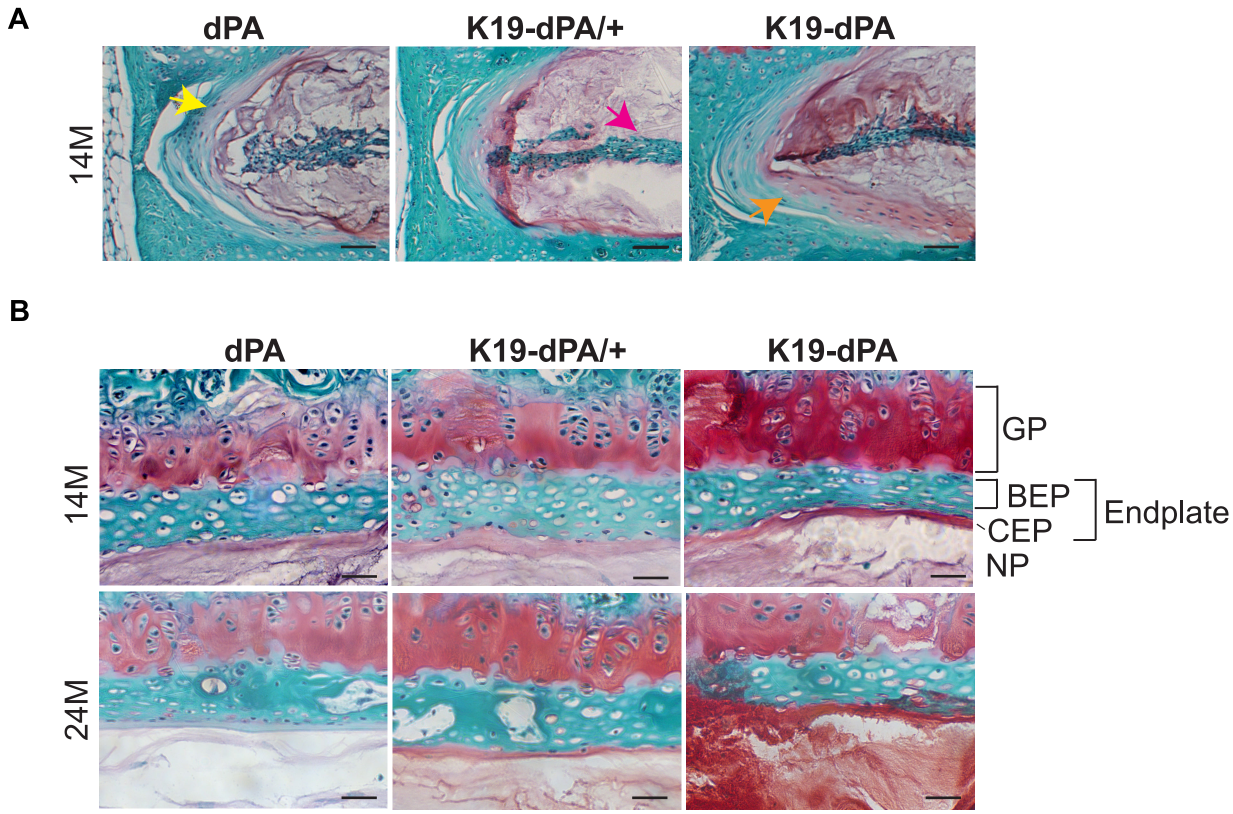

Supplementary Figure 1. (A) Safranin O/Fast Green staining of lumbar discs showing morphology of the NP and AF in 14M dPA, K19-dPA/+, and K19-dPA animals, yellow arrowhead indicates the AF distortion, pink arrowhead indicates the smaller NP cell band, and orange arrowhead indicates disruption at the AF interface, scale bar = 50 μm (B) Safranin O staining of the NP and endplates in 14M and 24M dPA, K19-dPA/+, and K19-dPA animals, scale bar = 25 μm.

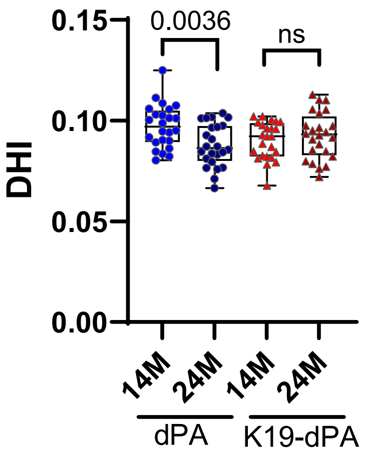

Supplementary Figure 2. Quantification of disc height index (DHI) calculated from reconstructed µCT images of hemi-sections of lumbar motion segments in 14-month and 24-month-old dPA and K19-dPA lumbar discs, n = 14M: 6 dPA (3 males, 3 females), 6 K19-dPA (4 males, 2 females) mice; 24M: 6 dPA (3 males, 3 females), 6 K19-dPA (3 males, 3 females) mice; 6 lumbar discs and 7 vertebrae/mouse were analyzed. Significance for quantitative measures was determined by using an unpaired t test with Welch's correction, as appropriate. Quantitative measurements represent the median with the interquartile range. ns = not significant.

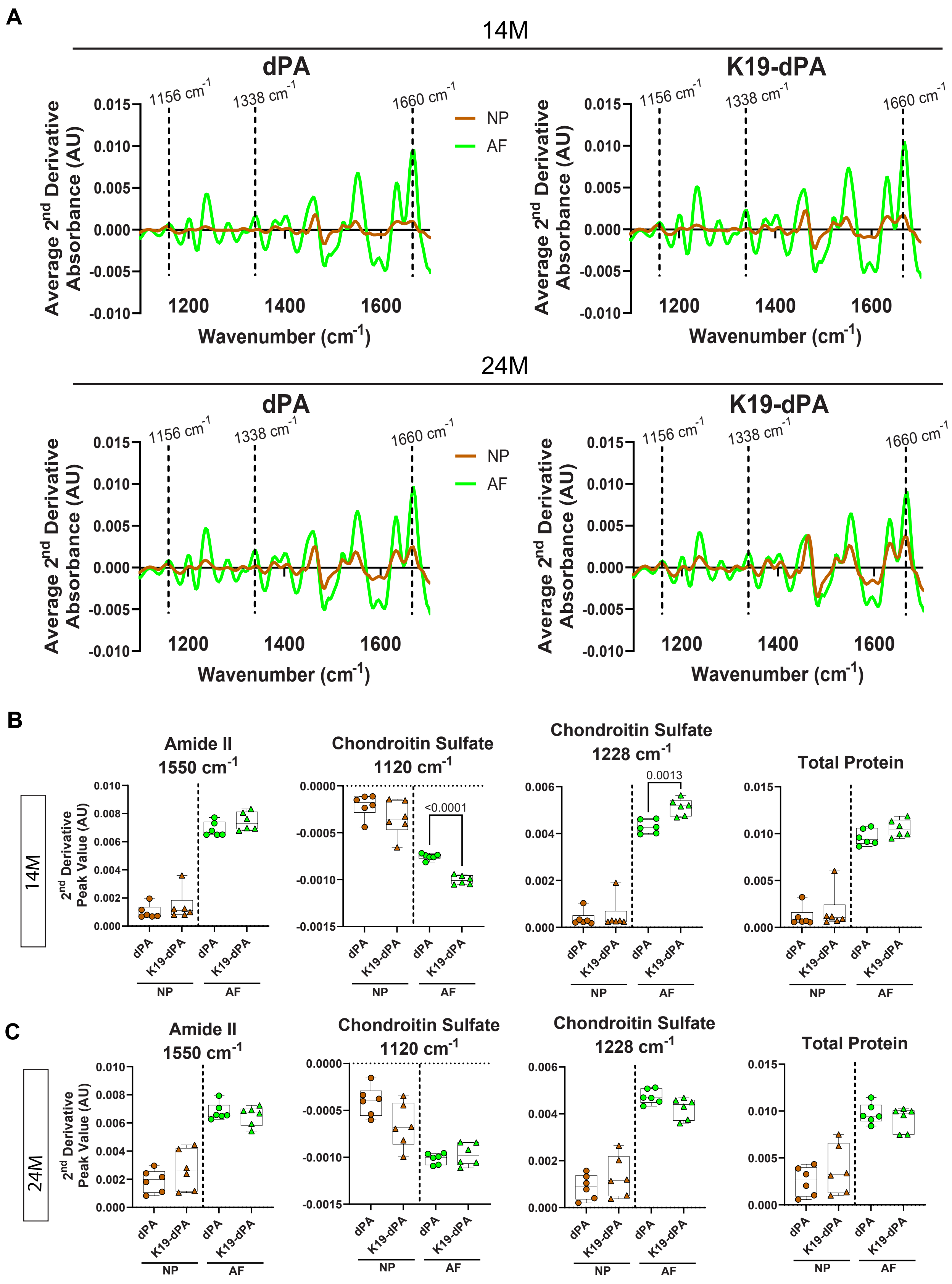

Supplementary Figure 3. (A) NP and AF superimposed average second derivative spectra, inverted for positive visualization from 14M and 24M dPA and K19-dPA NP mice. (B) Quantification of mean second derivative peaks associated with total protein (1660 cm−1), cell-associated proteoglycan (1156 cm−1), and AU = arbitrary units. ‘ n = 6 mice/genotype, 3 discs/mouse, 18 total discs/genotype. Significance for quantitative measures was determined by using Mann–Whitney U test or an unpaired t-test with Welch’s correction, as appropriate. Quantitative measurements represent the median with the interquartile range.

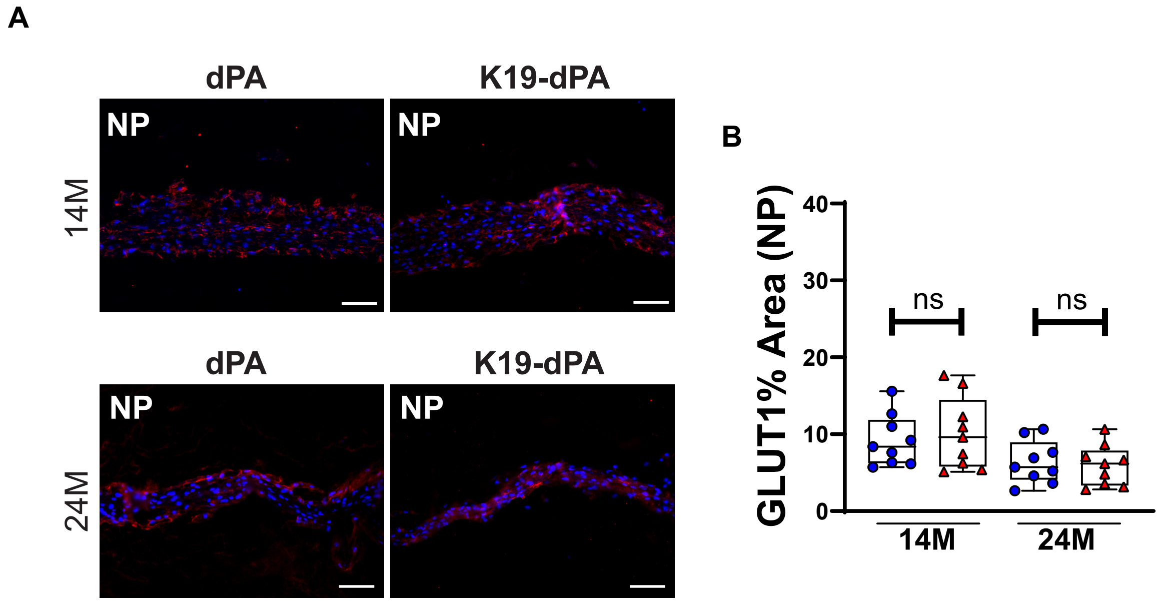

Supplementary Figure 4. (A) Quantitative immunofluorescent staining for glucose transporter 1 (GLUT1), in intervertebral discs of 14M and 24M dPA and K19-dPA lumbar discs, scale bar = 50 μm, *n* = 3 mice/genotype/time point, 3 discs/mouse. Significance was determined by unpaired t-test with Welch's correction. Quantitative measurements represent the median with the interquartile range. ns = not significant.

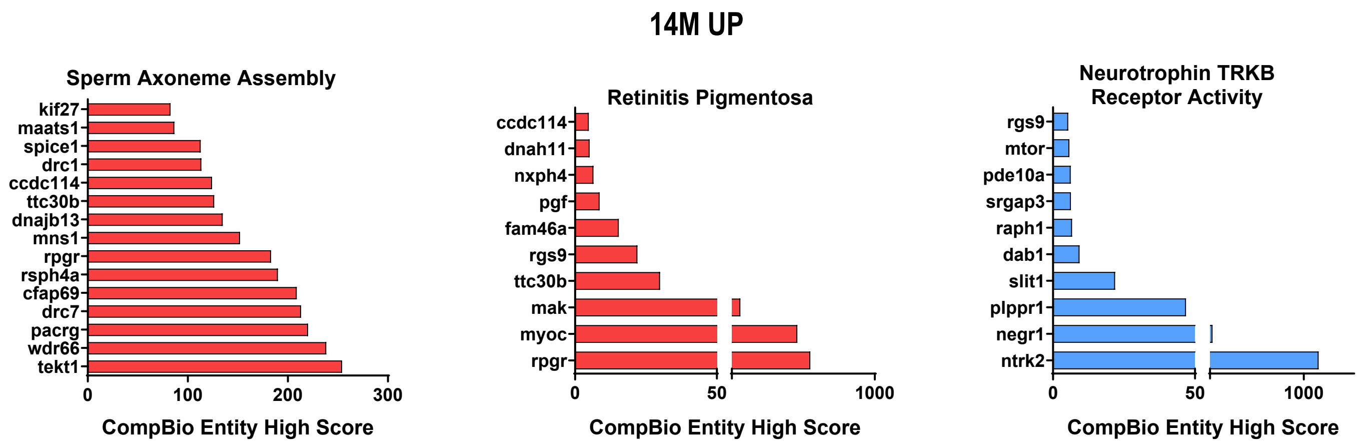

Supplementary Figure 5. 14-month upregulated DEGs from select central themes plotted based on their CompBio entity scores. n = 4 mice/genotype.

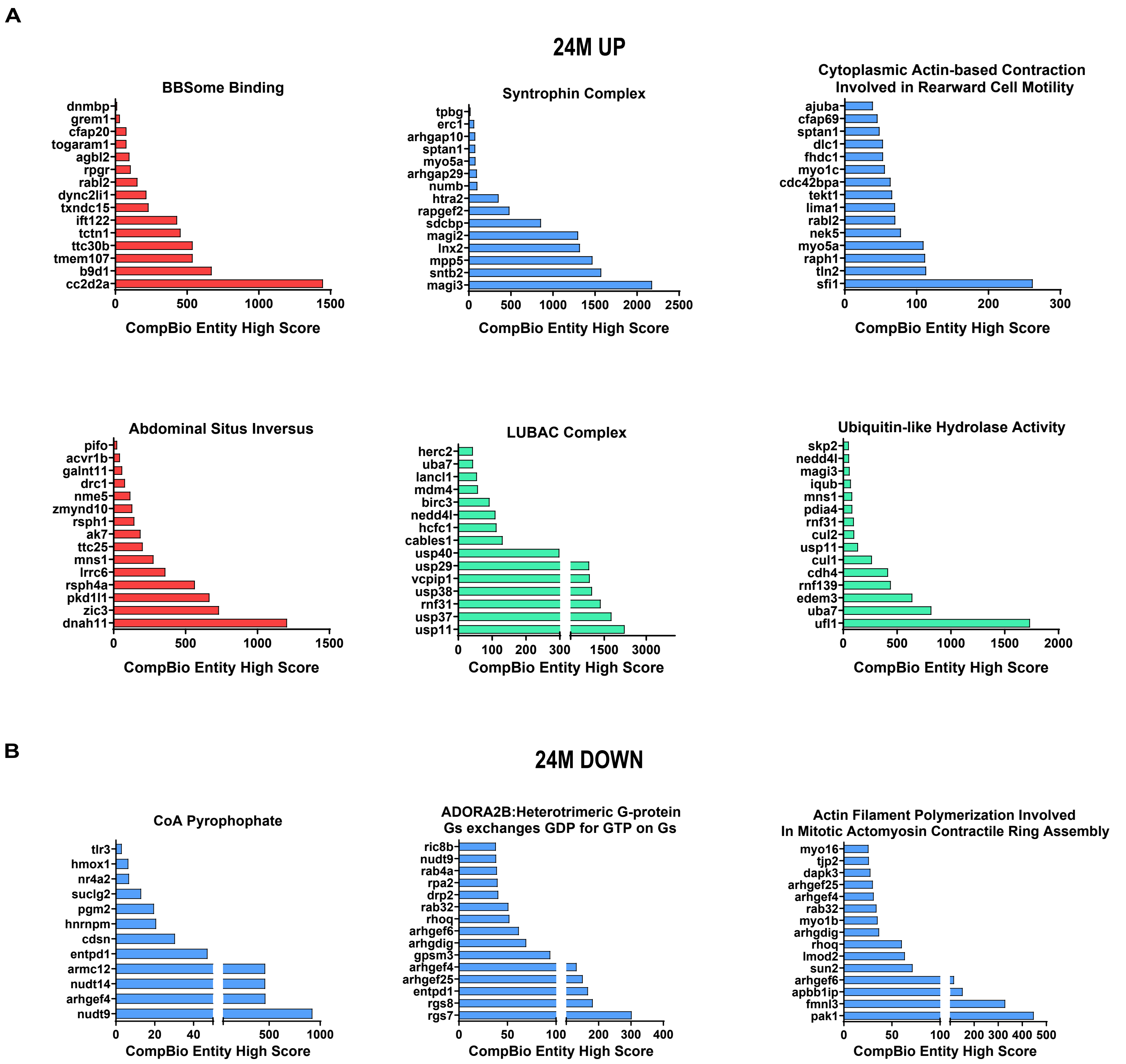

Supplementary Figure 6. (A) 24-month upregulated and (B) down-regulated DEGs from select central themes plotted based on their CompBio entity scores. n = 4 mice/genotype

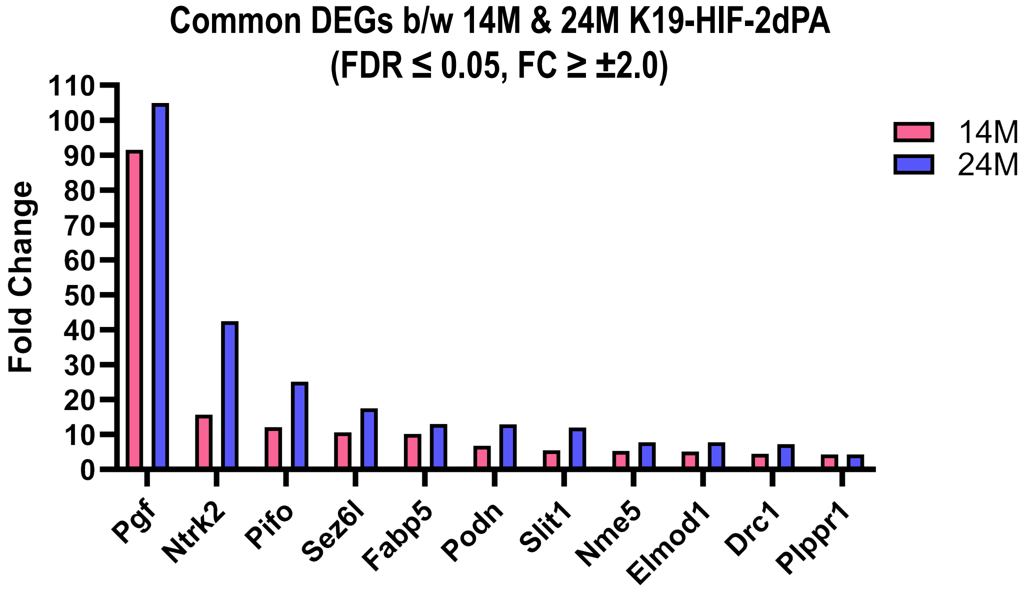

Supplementary Figure 7. (A) The highly enriched common DEGs in the NP between 14M and 24M K19-dPA mice with a fold change> ±2 and FDR < 0.05. n = 4 mice/genotype/time point.
